# 416. Impact of clinical diagnostic stewardship of respiratory cultures across a multicenter PICU collaborative

**DOI:** 10.1093/ofid/ofae631.130

**Published:** 2025-01-29

**Authors:** Anna Sick-Samuels, Daniel Kelly, Charlotte Woods-Hill, Abigail Arthur, Urmi Kumar, Danielle W Koontz, Anping Xie, Jill A Marsteller, Shaoming Xiao, Elizabeth Colantuoni, Aaron Milstone

**Affiliations:** Johns Hopkins University, Baltimore, MD; Boston Children's Hosptial, Boston, Massachusetts; Children's Hospital of Philadelphia, Philadelphia, PA; Johns Hopkins University School of Medicine, Baltimore, Maryland; Johns Hopkins University School of Medicine, Baltimore, Maryland; JHU SOM, Baltimore, Maryland; Johns Hopkins University, Baltimore, MD; Johns Hopkins Bloomberg School of Public Health, Baltimore, Maryland; Johns Hopkins University, Baltimore, MD; Johns Hopkins, Baltimore, MD; Johns Hopkins University, Baltimore, MD

## Abstract

**Background:**

Respiratory culture practices for critically ill patients with artificial airways vary across and within institutions. Over-testing contributes to unnecessary antibiotic treatment. This study aimed to evaluate the efficacy and safety of implementing clinical decision support to standardize indications of respiratory cultures across a multicenter PICU collaborative.

Monthly Respiratory Culture Rate per 100 Ventilator-Days Before and After Implementing Diagnostic Stewardship Programs

**Figure:**
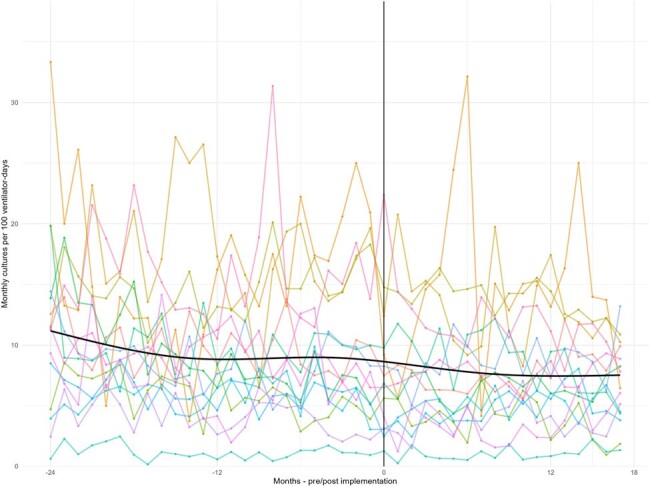

The monthly respiratory culture rate is shown for 15 pediatric intensive care units participating in BrighT STAR Respiratory, a multicenter diagnostic stewardship quality improvement collaborative, 24 months before and 18 months after implementing diagnostic stewardship programs. The mean monthly average rate over time was estimated using a smoothing spline.

**Methods:**

15 U.S. PICUs participated in the BrighT STAR Respiratory Collaborative, a prospective multicenter quality improvement program (QI) from 2019-2023. Each hospital executed local QI programs to improve respiratory culture practices, facilitated by the larger collaborative. The primary outcome was the monthly rate of respiratory cultures per 100 ventilator-days. Process and safety measures included the proportion of cultures repeated within 3 days, bronchoalveolar lavage (BAL) cultures per 100 ventilator-days, and ventilator-associated events (VAE) per 100 ventilator-days among 3 centers conducting this surveillance. We analyzed rates 24 months pre- and 18 months post-intervention using a Poisson generalized linear mixed model with random intercept for sites, robust variance estimates and adjustment for seasonality. Site leads surveyed local faculty for patient safety concerns.

**Results:**

Across 15 PICUs, the monthly average respiratory culture rate was 7.80 per 100 ventilator-days pre-implementation and 6.55 per 100 ventilator-days post-implementation, a 16% rate ratio (RR) reduction (95% confidence interval (CI) 0.78-0.90, P< 0.001). Cultures repeated within 3 days declined 22% from 13% to 10% (95%CI 0.68-0.89, P< 0.001). The BAL culture rate did not change from 0.67 to 0.72 (RR 1.07, 95% 0.85-1.34, P=0.57). The VAE rate did not change from 0.21 to 0.25 (RR 1.17, 95%CI 0.87-1.58, P=0.28). Among 2,525 inquiries to attendings about safety concerns, 2348 replied (93%); 2 concerns were reported (0.08%) regarding perceived delay to cultures with no resulting patient harm.

**Conclusion:**

Standardizing respiratory culture ordering practices may be an effective diagnostic stewardship approach for pediatric patients with artificial airways. We plan to assess impact on clinical outcomes and antibiotic use and to assess facilitators and barriers to implementation.

**Disclosures:**

**Aaron Milstone, MD, MHS**, Merck: Grant/Research Support

